# What are the optimal measures to identify anxiety and depression in people diagnosed with head and neck cancer (HNC): a systematic review

**DOI:** 10.1186/s41687-020-00189-7

**Published:** 2020-04-23

**Authors:** Chindhu Shunmugasundaram, Claudia Rutherford, Phyllis N. Butow, Puma Sundaresan, Haryana M. Dhillon

**Affiliations:** 1 University of Sydney, School of Psychology, Centre for Medical Psychology and Evidence-Based Decision-Making, Sydney, Australia; 2 University of Sydney, School of Psychology, Psycho-Oncology Cooperative Research Group, Sydney, Australia; 3 University of Sydney, School of Psychology, Quality of Life Office, Sydney, Australia; 4 University of Sydney, Sydney Nursing School, Cancer Nursing Research Unit (CNRU), Sydney, Australia; 5 Radiation Oncology Network, Western Sydney Local Health District, Sydney, Australia; 6 University of Sydney, Faculty of Medicine, Sydney Medical School, Sydney, Australia

**Keywords:** Anxiety, Depression, Psychological distress, Head and neck cancer, Systematic review, Patient reported outcome measures

## Abstract

**Background:**

A cancer diagnosis is potentially life-threatening, likely causing distress and uncertainty, which may be psychologically debilitating. Depression and anxiety are commonly underdiagnosed and undertreated in cancer patients. Head and neck cancer (HNC) patients face particular challenges that may contribute to distress. This review aims to: i) identify patient reported outcome measures (PROMs) designed to assess anxiety and depression in HNC; and ii) determine their suitability for use in research and clinical practice to screen patients.

**Methods:**

We searched five electronic databases between July 2007 to July 2019 for studies assessing anxiety and depression in HNC patients. Searches were limited to this period to account for advances in cancer treatment. Records were screened for eligibility by one reviewer and 10% cross-checked by a second across all stages of the review. In addition to the electronic searches, PROM databases were searched for additional measures of anxiety and depression. All retrieved PROMs were mapped against Diagnostic and Statistical Manual-5 criteria for anxiety and depression to assess content coverage. Then, their psychometric properties appraised against the COSMIN checklist.

**Results:**

Electronic searches identified 98 records, from which five anxiety and eight depression measures were retrieved. PROM database searches retrieved an additional four anxiety and four depression measures; a total of nine anxiety and 12 depression measures were appraised. Content coverage of anxiety measures ranged from 50% to 75% and depression measures from 42% to 100%. Demonstration of psychometric properties against COSMIN criteria ranged from 57% to 71% for anxiety measures (three PROMs > 70%) and from 29% to 86% for depression measures (nine PROMs > 70%). Three anxiety and seven depression measures had established clinical cut-offs in cancer populations.

**Conclusions:**

The Patient Health Questionnaire-9, Zung Self-rating Depression and Zung Self-rating Anxiety Scales demonstrated good content coverage along with excellent psychometric properties, and thus were considered the most suitable PROMs to assess psychological distress in HNC populations. It is important to have PROMs assessing psychological distress that capture a comprehensive set of subjective symptoms. The identified PROMs will help researchers and health professionals in clinical-decision making, thereby potentially improving quality of life in HNC patients.

## Background

Diagnosis of any life-threatening illness can result in multiple emotional and psychological reactions. A cancer diagnosis can evoke existential distress, and necessitate an acceptance of uncertainty. Of all people with cancer, those diagnosed with head and neck cancer (HNC) experience high rates of depression and anxiety during and after treatment [1, 2], due to the location of the cancer and its impact on appearance and critical functions. HNCs affect body parts visible to the outside world and are responsible for the most fundamental, life sustaining functions such as speech, eating, swallowing and breathing. The physical effects of these cancers may result in social withdrawal and poor emotional expression rendering HNC patients more prone to depression or anxiety than those with other cancers [3–8].

Depression and anxiety in people with cancer are commonly underdiagnosed and undertreated despite health professionals knowing the prevalence [9]. Psychological morbidity could impact patients’ Health Related Quality of Life (HRQoL), limit their social activities, increase their hospital stay, delay their return to work, and influence their ability to care for themselves [10, 11]. Indeed, one consistent factor impacting HRQoL is clinical depression [12, 13]. If severe, depression can diminish cancer patients’ decision making capacity related to their treatment, resulting in reduced acceptance of adjuvant therapies and more unplanned breaks in treatment, compromising survival [14]. Studies have also shown that depressed cancer patients are more likely to have disease recurrence and poorer survival [15], making it critically important to recognise and treat depression when it occurs.

Screening for and diagnosing anxiety and depression have been priorities for the psycho-oncology community for at least a decade, with calls for distress to be promoted as the sixth vital sign [16]. In addition to screening, assessing diagnostic criteria are critically important. The main diagnostic criteria of depression, according to DSM-5 are disturbed sleep and appetite, fatigue, depressed mood, agitation, difficulty concentrating, self-esteem issues and suicidal thoughts [17]. While the main diagnostic criteria of clinical anxiety are: constant worry, restlessness, panic, worry, nervousness, disturbing thoughts, poor concentration, irritability, fatigue or loss of energy, muscle tension and sleep disturbances [17].

Prevalence rates of clinical levels of depression and anxiety vary by cancer type, its severity, and impact of treatment on structural and functional deficits [18, 19]. Apart from cancer and its treatment, other factors including personality traits, coping skills, pain, prognosis, substance usage or dependence, body image disturbance, previous history of psychiatric illness and social support may be related to depression in patients with HNC [10]. The most common factors triggering anxiety during and after HNC treatment are fear of cancer recurrence, reduced communication abilities, dysphagia, changes in appearance, and adapting to dysfunction [20]. A study of surgically treated HNC patients described high levels of anxiety and depression with dominating anxiety symptoms when assessed using the Hospital Anxiety and Depression scale [21]. Studies report a prevalence of 25–33% for anxiety and depression in HNC populations post-treatment [22, 23].

There is strong evidence that psychosocial interventions improve psychological outcomes in cancer patients with varying cancer diagnoses. However, to effectively improve outcomes, interventions must be tailored to the target populations. To design tailored interventions and evaluate them, appropriate measures are necessary. Patient reported outcome measures (PROMs) assessing anxiety and depression in HNC patients should have items that are sensible, appropriate and relevant to that population. However, while a number of reviews have collated and summarised measures of anxiety and depression in cancer settings, none has considered their use with HNC patients and survivors. Hence, we conducted a systematic review of anxiety and depression PROMs in HNC.

Our specific aims were to:

1. Identify available PROMs assessing anxiety and depression in the HNC setting;

2. Map items against anxiety and depression criteria adapted from DSM-5 to assess conceptual coverage;

3. Appraise their psychometric properties to determine clinically robust and disease-specific PROMs able to screen for and detect anxiety and depression in HNC populations.

## Methods

This study was part of a larger systematic review registered under ID CRD42018080677 with PROSPERO. A search was carried out using five online databases - CINAHL, Medline, EMBASE, Web of Science and PsycInfo - to locate all studies relevant to the aims of this review. The search strategy included a broad set of terms for ‘anxiety’, ‘depression, and ‘head and neck cancer’ developed by the authors for Medline and PsycInfo (via Ovid) and adapted for other databases (see Additional file 1 for search strategy developed for Medline and PsycInfo via Ovid). Searches were limited to studies reported between July 2007 to January 2020 (current) to reflect treatment advances in that decade. Language restrictions were applied, and only studies published in English were screened. To supplement electronic searches, we searched online PROM databases (PROQOLID, Psycho-oncology database (POD) and Grid-enabled Measures Database (GEM)) for additional measures of anxiety and depression.

### Study selection

Titles and abstracts from retrieved studies were screened against the following inclusion and exclusion criteria by one reviewer (CS).

Inclusion criteria:

i. Papers including at least one PROM assessing either anxiety, depression, or both.
ii. Sample included patients or survivors of any type of HNC (oral cavity, hypopharyngeal, oropharyngeal, laryngeal, nasal and sinus gland, salivary gland and nasopharyngeal) except thyroid, either as sole or mixed tumour groups (where HNC was included as a sub-group with results reported separately), aged 18 years or older.
iii. Responses obtained directly from HNC patients about their anxiety and depression (screening, extent, or severity)
iv. Primary research

We excluded systematic reviews, conference abstracts, letters to the editor, discussion papers, notes, case studies and conference proceedings, and non-English papers. A second reviewer (HD) screened 10% of all titles, abstracts and full text of articles. Measures from online PROM databases were included if they assessed either anxiety, depression or both in cancer patients and reports of their development and validation were available.

### Extraction

Study title, aims, rationale, PROM(s) used, sample demographics and characteristics, study design, methods, results, limitations and conclusions were extracted by one reviewer. A second reviewer extracted 10% of all full texts and reviewed all extractions for errors and accuracy.

### Analysis

The analysis consisted of two phases – content mapping and appraisal of psychometric properties of identified PROMs. Items from identified PROMs were mapped to DSM-5 criteria for depression and anxiety, as DSM-5 is a widely used and recognised authoritative guide containing descriptions, symptoms and criteria for diagnosing mental disorders such as depression and anxiety. DSM-5 is an evidence-based manual developed from scientific research and collective knowledge of clinicians and experts in medical and mental health disciplines [17]..

Mapping was done by three reviewers (CS, HD and PB) to ensure accuracy of content mapping and to assess the extent of content coverage. A few minor disagreements were resolved through group discussion until consensus was achieved. Percentage of content coverage was calculated for all PROMs to determine their relevance to anxiety and depression.

All PROMs’ psychometric properties were assessed against the COnsensus-based Standards for the selection of health Measurement INstruments (COSMIN) checklist [24] including: item generation, item reduction, validity, reliability, hypothesis testing, responsiveness, clinical cut-off and clinical cut-off for cancer populations.

## Results

**Fig. 1:**
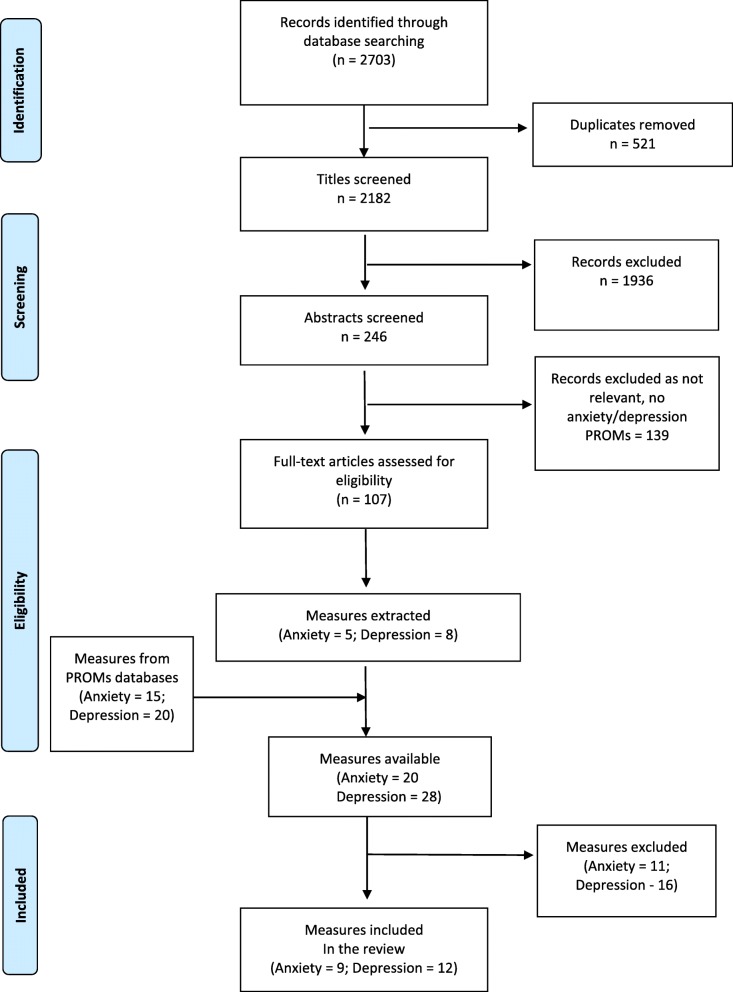
Prisma flow diagram describing unmet needs measures search

A total of 2703 articles were retrieved from electronic searches. After screening for duplicates and eligibility, 107 studies were retained. A total of five anxiety and eight depression measures were identified across these studies. Online PROM database searches identified an additional 140 anxiety and 114 depression measures. After screening against our eligibility criteria, 15 anxiety and 20 depression measures were retained. In total, from included studies and online PROMs databases, a total of nine anxiety and 12 depression measures were retained for further analysis (Fig. 1 details the PRISMA flow diagram).

All PROMs other than QIDS-SR, MASQ, Duke-AD, MDI and CDS had been tested for relevance to patients with cancer [25–39]. Ten of the 21 PROMs identified had established clinical cut off scores for anxiety and/or depression in cancer populations. Content mapping of each anxiety and depression PROM are summarized below and presented in Tables 1 and 2 respectively. Appraisals of their development and psychometric validation characteristics are summarized below and presented in Tables 3 and 4 respectively.

**Table 1:** Patient reported outcome measures of anxiety: content mapping

Anxiety (adapted from DSM-5)	HADS	STAI	SAS	BAI	MASQ	DASS 21	Duke-AD	BSI-18	GAD-7
Restlessness	✓	✓	✓	✓	✓	✓	✓	✓	✓
Fatigue			✓			✓	✓		
Concentration		✓				✓	✓		
Irritability		✓					✓		✓
Muscle tension	✓	✓	✓	✓	✓			✓	✓
Sleep disturbances			✓				✓		
Disturbing thoughts	✓	✓	✓	✓	✓	✓		✓	✓
Worry	✓	✓	✓	✓	✓	✓		✓	✓
*Number domains covered*	4	6	6	4	4	5	5	4	5

✓ **-** Present

*HADS* Hospital anxiety and depression scale; *STAI* State trait anxiety inventory, *SAS* Zung self-rating anxiety scale, *BAI* Beck Anxiety inventory, *MASQ* Mood and anxiety symptom questionnaire, *DASS 21* Depression anxiety stress scale −21, *Duke-AD* Duke anxiety depression scale, *BSI-18* Brief symptom inventory − 18, *GAD-7* Generalized anxiety disorder – 7

**Table 2:** Patient reported outcome measures of depression: content mapping

Depression (adapted from DSM-5)	HADS	QIDS-SR	SDS	BDI	CES-D	PHQ-9	GDS-SF	BSI-18	Duke-AD	MDI	CDS	DASS 21
Depressed mood	✓	✓	✓	✓	✓	✓	✓	✓	✓	✓	✓	✓
Lowered interest or pleasure in all	✓	✓	✓	✓	✓	✓	✓	✓		✓	✓	✓
Change in weight		✓	✓	✓							✓	
Change in sleep		✓	✓	✓	✓	✓			✓	✓	✓	
Psychomotor agitation/restlessness	✓	✓	✓			✓	✓	✓	✓	✓	✓	✓
Fatigue/Loss of energy	✓	✓	✓	✓	✓	✓	✓	✓	✓	✓	✓	
Feelings of worthlessness or guilt		✓	✓	✓	✓	✓	✓	✓		✓	✓	✓
Indecisiveness/diminished ability to think		✓	✓	✓			✓				✓	
Suicidal ideation		✓	✓	✓		✓	✓	✓		✓	✓	
Concentration		✓			✓	✓	✓		✓	✓	✓	
Low self-esteem	✓	✓	✓	✓	✓	✓	✓	✓		✓	✓	✓
*Number domains covered*	5	11	10	9	7	9	9	7	5	9	11	5

✓ **-** Present

*HADS* Hospital anxiety and depression scale, QIDS-SR Quick inventory of Depressive Symptomatology self-report, SDS Zung self-rating depression scale, BDI Beck Depression inventory, CES-D Center for Epidemiologic Studies Depression Scale, PHQ-9 Patient Health Questionnaire −9, GDS-SF Geriatric Depression Scale – Short form, BSI-18 Brief symptom inventory − 18, Duke-AD Duke anxiety depression scale, MDI Major depression inventory, CDS Carroll Rating Scale for Depression, DASS 21 Depression anxiety stress scale − 21

**Table 3:** Patient reported outcome measures of anxiety: measurement properties

	Method^a^	HADS	STAI	SAS	BAI	MASQ	DASS 21	Duke-AD	BSI-18	GAD-7
*Item generation*	Literature	✓	✓	✓	✓	✓	✓	✓	✓	✓
	Patient/person interviews			✓					✓	
	Clinician interviews/Expert opinion								✓	
*Item reduction*	Missing data for summary scores									
	Missing item data									
	Factor Analysis	✓	✓		✓	✓	✓		✓	✓
*Psychometric analyses*	Cronbach’s α	✓	✓	✓	✓	✓	✓	✓	✓	✓
	Test-retest reliability	✓	✓	✓	✓		✓	✓	✓	✓
	Content validity	✓	✓		✓	✓	✓		✓	✓
	Item total correlations	✓	✓		✓	✓	✓		✓	✓
	Convergent/discriminant (or divergent) validity	✓		✓	✓	✓	✓	✓	✓	✓
	Hypothesis testing	✓		✓		✓	✓	✓		✓
	Translated into other languages	✓	✓	✓	✓		✓	✓	✓	
*Cut off points*	Responsiveness	✓	✓	✓	✓		✓	✓	✓	
	Clinical cut-off	✓	✓	✓	✓	✓	✓	✓	✓	✓
	Clinical cut-off for cancer	✓					✓		✓	

*HADS* Hospital anxiety and depression scale, STAI State trait anxiety inventory, SAS Zung self-rating anxiety scale, BAI Beck Anxiety inventory, MASQ Mood and anxiety symptom questionnaire, DASS 21 Depression anxiety stress scale −21, Duke-AD Duke anxiety depression scale, BSI-18 Brief symptom inventory − 18, GAD-7 Generalized anxiety disorder – 7

^a^Criteria based on the COSMIN checklist; ✓ - Present

**Table 4:** Patient reported outcome measures of depression: measurement properties

	Method^a^	HADS	QIDS-SR	SDS	BDI	CES-D	PHQ-9	GDS-SF	BSI-18	DUKE-AD	MDI	CDS	DASS 21
*Item generation*	Literature	✓	✓	✓	✓	✓	✓	✓	✓		✓	✓	✓
	Patient/person interviews	✓	✓	✓	✓	✓	✓	✓	✓		✓		
	Clinician interviews/Expert opinion		✓	✓			✓	✓	✓				
*Item reduction*	Missing data for summary scores												
	Missing item data												
	Factor Analysis	✓	✓	✓	✓	✓	✓	✓	✓		✓	✓	✓
*Psychometric analyses*	Cronbach’s α	✓	✓	✓	✓	✓	✓	✓	✓	✓	✓	✓	✓
	Test-retest reliability	✓		✓	✓	✓	✓	✓	✓	✓			✓
	Content validity	✓	✓	✓	✓	✓	✓	✓	✓		✓		✓
	Item total correlations	✓ ✓	✓	✓	✓	✓	✓		✓		✓		✓
	Convergent/discriminant (or divergent) validity	✓	✓	✓	✓	✓	✓	✓	✓	✓	✓	✓	✓
	Hypothesis testing	✓	✓	✓	✓	✓	✓	✓		✓			✓
	Translated into other languages	✓	✓	✓	✓	✓	✓	✓	✓	✓			✓
*Cut off points*	Responsiveness	✓	✓	✓	✓	✓	✓	✓	✓	✓	✓		✓
	Clinical cut-off	✓	✓	✓	✓	✓	✓	✓	✓	✓	✓		✓
	Clinical cut-off for cancer	✓		✓	✓	✓	✓		✓				✓

*HADS* Hospital anxiety and depression scale, *QIDS-SR* Quick inventory of Depressive Symptomatology self-report, *SDS* Zung self-rating depression scale, *BDI* Beck Depression inventory, *CES-D* Center for Epidemiologic Studies Depression Scale, *PHQ-9* Patient Health Questionnaire −9, *GDS-SF* Geriatric Depression Scale – Short form, *BSI-18* Brief symptom inventory −18, *Duke-AD* Duke anxiety depression scale, MDI Major depression inventory, *CDS* Carroll Rating Scale for Depression, *DASS 21* Depression anxiety stress scale −21

^a^Criteria based on the COSMIN checklist; ✓ - Present

More data on which PROMs had been used in HNC settings, the reasons for exclusion and final selection are presented in Table 5.

**Table 5:** PROMs used in HNC studies, reasons for inclusion and exclusion

PROMs	Used in HNC studies?	Reason for exclusion	Reason for selection
Anxiety PROMs			
HADS	✓	Average content coverage	
STAI	✓	Failed to differentiate participants with or without anxiety disorders	
SAS	✓		Excellent content coverage and good psychometrics
BAI	✓	Average content coverage	
MASQ	No	Average content coverage	
DASS 21	✓	Average content coverage	
Duke-AD	No	Average content coverage	
BSI-18	✓	Average content coverage	
GAD-7	✓	Average content coverage	
Depression PROMs			
HADS	✓	Poor content coverage	
QIDS-SR	✓	No clinical cut-off for cancer patients	
SDS	✓		Excellent content coverage and psychometrics
BDI	✓	Items on body image and hypochondriasis which could confound with effects from treatment	
CES-D	✓	Average content coverage	
PHQ-9	✓		Excellent content coverage and psychometrics
GDS-SF	✓	No clinical cut-off for cancer patients	
BSI-18	✓	Average content coverage	
Duke-AD	No	Poor content coverage	
MDI	No	No clinical cut-off for cancer patients	
CDS	No	Poor psychometrics	
DASS 21	✓	Poor content coverage	

✓ **-** Yes

*HADS* Hospital anxiety and depression scale, *STAI* State trait anxiety inventory, SAS Zung self-rating anxiety scale, *BAI* Beck Anxiety inventory, *MASQ* Mood and anxiety symptom questionnaire, DASS 21 Depression anxiety stress scale −21, *Duke-AD* Duke anxiety depression scale, *BSI-18* Brief symptom inventory −18, *GAD-7* Generalized anxiety disorder – 7

*QIDS-SR* Quick inventory of Depressive Symptomatology self-report, *SDS* Zung self-rating depression scale, *BDI* Beck Depression inventory, *CES-D* Center for Epidemiologic Studies Depression Scale, *PHQ-9* Patient Health Questionnaire −9, *GDS-SF* Geriatric Depression Scale – Short form, *MDI* Major depression inventory; *CDS* Carroll Rating Scale for Depression

### Anxiety

Of the nine anxiety measures, five demonstrated average content coverage when mapped against DSM-5 criteria for anxiety (Table 1) and five demonstrated adequate psychometric properties (Table 3). All nine anxiety measures identified are discussed below to provide sufficient information to enable each measure to be considered by readers.

### Hospital anxiety and depression scale (HADS)

The HADS is a 14-item self-report measure with seven items measuring anxiety [40, 41]. It has been specifically designed for use in the in-patient setting with people who are physically ill, thus it excludes symptoms of anxiety and/or depression that may reasonably be thought associated with being physically unwell. Cronbach’s alpha for HADS was 0.83 [41, 42]. HADS has established clinical cut-off scores for general patients with clinical anxiety and cancer patients [43], making it easier for administration, scoring and diagnosis. When mapped against DSM-5 criteria for anxiety, HADS covered only 50% of content relevant to anxiety and was missing items assessing fatigue, concentration, irritability, and sleep disturbances.

### State trait anxiety inventory (STAI)

The STAI is a 40-item self-report anxiety measure with 20 items measuring state anxiety and 20 items measuring trait anxiety [44]. STAI assesses the intensity of a person’s anxious feelings and has demonstrated a Cronbach’s alpha of 0.89 [44–46]. Cut-off points for each of the following populations have been established: general patients with clinical anxiety, a psychiatric sample, chronically ill patients, and patients before and after surgery (not restricting to any illness) [46–48]. When mapped against DSM-5 criteria for anxiety, STAI covered 75% of content, failing to include items examining fatigue and sleep disturbances.

### Zung self-rating anxiety scale (SAS)

Zung Self-Rating Anxiety Scale is a self-rated measure with 20 items [49]. It assesses affective and somatic symptoms of anxiety making it a measurement of anxiety as a clinical entity. SAS has a Cronbach’s alpha of 0.82 [49–51]. Cut-off points for anxiety in non-clinical populations and those with a clinical diagnosis have been established [52]. When mapped against DSM-5 criteria for anxiety, SAS covered 75% content but did not contain items examining concentration and irritability.

### Beck’s anxiety inventory (BAI)

Beck’s Anxiety Inventory is a 21-item self-rating symptom measure to detect the severity of anxiety in a population with psychiatric problems [53]. A Cronbach’s alpha of 0.92 demonstrated internal consistency [53, 54]. Cut-off points to detect clinical anxiety have been established but no literature supports clinical cut-off points for anxiety in cancer patients or chronically ill patients [55]. When mapped against DSM-5 criteria for anxiety, BAI had 50% content coverage, missing items examining sleep disturbance, irritability, concentration and fatigue.

### Mood and anxiety symptom questionnaire (MASQ)

Mood and Anxiety Symptom Questionnaire is a 90-item self-report measure developed to assess depression and anxiety symptoms ([56, 57]; Watson D, Clark LA: The mood and anxiety symptom questionnaire, Unpublished). The Cronbach’s alpha of MASQ ranged from 0.78 to 0.93 ([58]; Watson D, Clark LA: The mood and anxiety symptom questionnaire, Unpublished). Clinical cut-off points have been established for non-clinical samples but not for cancer populations [58]. When mapped against DSM-5 criteria for anxiety, MASQ had only 50% content coverage, missing items examining sleep disturbance, irritability, concentration and fatigue.

### Depression anxiety stress scale 21 (DASS 21)

DASS 21 is a 21-item self-report measure assessing the intensity of negative emotional states such as depression, anxiety and stress [35]. A Cronbach’s alpha of 0.84 for the anxiety construct was achieved, indicating good internal consistency [35, 59, 60]. In DASS-21, cut-off points for clinical patients, cancer patients, and non-clinical populations have been established separately [61]. When mapped against DSM-5 criteria for anxiety, DASS-21 covered 63% of content, missing items assessing irritability, sleep disturbances and muscle tension.

### Duke anxiety depression scale (Duke-AD)

Duke AD is a seven-item self-report measure used to assess anxiety and depression [62]. Duke AD’s Cronbach’s alpha is 0.69 [63]. Cut-off points for primary care patients and non-clinical subjects have been established [62, 63]. When mapped against DSM-5 criteria for anxiety, Duke-AD covered 63% of content, missing items assessing muscle tension, disturbing thoughts and worry.

### Brief symptom inventory – 18 (BSI-18)

Brief Symptom Inventory is an 18-item self-report measure used to measure the psychological distress of psychiatric and medical patients and of non-clinical samples [64]. Cronbach’s alpha of all domains in BSI-18 ranged from 0.71 to 0.85 [65]. Cut-off points for clinical patients, mixed cancer groups, survivors, palliative patients and healthy populations have been separately established [64, 65]. When mapped against DSM-5 criteria for anxiety, BSI-18 covered 50% content, missing items measuring fatigue, concentration, irritability and sleep disturbance.

### Generalized anxiety disorder – 7 (GAD – 7)

GAD-7 is a seven-item self-report measure used to assess generalized anxiety disorder and to measure the extent of symptom severity [66]. Cronbach’s alpha of GAD-7 has been demonstrated as 0.92 [66]. Cut-off points have been established for patients with clinical anxiety or generalized anxiety disorder and non-clinical populations [66, 67]. When mapped against DSM-5 criteria for anxiety, GAD-7 had 63% content coverage, missing items assessing fatigue, concentration and muscle tension.

### Depression

Of the 12 depression measures, seven demonstrated average to good content coverage when mapped against DSM-5 criteria for depression (Table 2) and nine demonstrated adequate psychometric properties (Table 4). All 12 depression measures identified are discussed below to provide sufficient information to enable each measure to be considered by readers.

### Hospital anxiety and depression scale (HADS)

The HADS is a 14-item self-report measure with seven items measuring depression [40, 41]. Cronbach’s alpha for HADS was 0.83 [41, 42]. HADS has established clinical cut-off scores for general patients with clinical depression and cancer patients [43], allowing for easy administration, scoring and diagnosis. When mapped against DSM-5 criteria for depression, HADS covered only 42% of content relevant to depression, not including items assessing change in weight, change in sleep, feelings of worthlessness/guilt, indecisiveness, suicidal ideation, concentration and appetite.

### Quick inventory of depressive symptomatology self-report (QIDS-SR)

The QIDS-SR is a 16-item self-report measure used to assess the severity of depressive symptoms [68]. The Cronbach’s Alpha of the measure was reported to be 0.86 [68]. Clinical cut-off points have been established for non-clinical subjects and patients with major depressive disorder [68, 69]. When mapped against DSM-5 criteria for depression, QIDS-SR had 100% content coverage, including all items needed to assess depression.

### Zung self-rating depression scale (SDS)

Zung Self-Rating Depression Scale is a 20-item measure developed to assess depression in patients with depressive disorders [70, 71]. The internal consistency of the measure ranged between 0.88 and 0.93 [72]. Clinical cut-off points have been established for non-clinical populations, patients with depression and cancer patients [72, 73]. When mapped against DSM-5 criteria for depression, the SDS had 92% content coverage, missing out an item assessing concentration.

### Beck’s depression inventory (BDI)

Beck’s Depression Inventory is a 21-item self-report measure developed to measure the severity of depression [74, 75]. The measures’ Cronbach’s Alpha ranged from 0.92 to 0.93 [74, 76]. Clinical cut-off points have been established for psychiatric outpatients, medical patients, non-clinical populations and cancer patients [74, 76, 77]. When mapped against DSM-5 criteria for depression, the BDI had 83% content coverage, lacking items assessing psychomotor agitation/restlessness and concentration.

### Center for Epidemiologic Studies Depression Scale (CES-D)

CES-D is a 20-item self-report measure developed to assess the frequency and severity of depressive symptoms [78]. Cronbach’s Alpha of CES-D ranged from 0.84–0.85 [78–80]. Clinical cut-off points have been established for non-clinical, psychiatric, and cancer and cancer survivor populations [80, 81]. When mapped against DSM-5 criteria for depression, the CES-D had only 67% content coverage, not consisting of items measuring change in weight, psychomotor agitation/restlessness, indecisiveness and suicidal ideation.

### Patient health questionnaire – 9 (PHQ-9)

PHQ-9 is a nine-item self-report measure developed to assess depressive disorders, functional impairment and psychosocial stressors [82]. Cronbach’s Alpha of PHQ-9 was demonstrated to be 0.89 [82]. Clinical cut-off points have been established for non-clinical and cancer populations [83]. When mapped against DSM-5 criteria for depression, the PHQ-9 had 83% content coverage, lacking items measuring change in weight and indecisiveness.

### Geriatric depression scale -Short form (GDS-SF)

GDS-SF is a 15-item self-report measure developed to measure depressive symptoms in the geriatric populations [84]. Cronbach’s alpha was reported to range between 0.74–0.86 [85]. Clinical cut-off points have been established for older adults [86]. When mapped against DSM-5 criteria for depression, the GDS-SF had 75% content coverage, lacking items measuring change in weight, change in sleep and appetite.

### Brief symptom inventory 18 (BSI-18)

Brief Symptom Inventory is an 18-item self-report instrument used to measure the psychological distress of psychiatric and medical patients and of non-clinical populations [64]. Cronbach’s alpha for all domains in BSI-18 ranged from 0.71 to 0.85 [65]. Cut-off points have been established for clinical patients, mixed cancer groups, survivors, palliative patients and healthy populations [64, 65]. When mapped against DSM-5 criteria for depression, BSI-18 had only 58% content coverage, lacking items measuring change in weight, change in sleep, indecisiveness, concentration and appetite.

### Duke-anxiety depression scale (Duke-AD)

Duke AD is a seven-item self-report measure used to assess anxiety and depression [62]. Duke AD’s Cronbach’s alpha is 0.69 [63]. Cut-off points have been established for primary care patients and non-clinical subjects [62, 63]. When mapped against DSM-5 criteria for depression, Duke-AD covered only 42% of content, missing items measuring feelings of worthlessness or guilt, indecisiveness, suicidal ideation, low self-esteem and appetite.

### Major depression inventory (MDI)

Major Depression Inventory is a 10-item self-report measure developed to assess the severity of depressive states [87]. Cronbach’s Alpha of MDI was reported to be 0.90 [88]. Clinical cut-off points have been established for patients with major depressive disorder and other depressive states [89]. When mapped against DSM-5 criteria for depression, MDI covered 83% of content, lacking items measuring change in weight and indecisiveness.

### Carroll rating scale for depression (CDS)

Carroll Rating Scale for Depression is a 52-item self-report measure developed to assess behavioural and somatic manifestations of depression in psychiatric patients [90]. Cronbach’s Alpha of CDS was found to be 0.80 [90]. Clinical cut-off points have been established for patients with clinical depression [90]. When mapped against DSM-5 criteria for depression, CDS covered 92% of content, missing items assessing appetite.

### Depression anxiety stress scale 21 (DASS 21)

DASS 21 is a 21-item self-report measure used to assess the intensity of negative emotional states such as depression, anxiety and stress [35]. Depression subscale Cronbach’s alpha was reported to be 0.94 [35, 59, 60]. Cut-off points have been established for clinical patients, cancer patients, and non-clinical populations [61]. When mapped against DSM-5 criteria for depression, DASS 21 covered only 42% of content, lacking items covering change in weight, change in sleep, fatigue/loss of energy, indecisiveness, suicidal ideation, concentration and appetite.

All PROMs identified other than MASQ, Duke-AD, MDI and CDS (measures obtained from PROMs databases) had been used in HNC populations. Based on content mapping and appraisal of psychometric properties, SAS, SDS and PHQ-9 were considered most suitable for usage in HNC population to assess anxiety and depression.

## Discussion

Optimal outcomes for cancer patients necessitate that not only disease specific and treatment morbidity related outcomes are addressed but also HRQoL outcomes as reported by the patients through use of appropriate PROMs. Given the prevalence of anxiety and depression in cancer patients, in particular HNC, adopting measures of anxiety and depression appropriate for use in the HNC populations is critical for accurate detection of and intervention for anxiety and depression. We identified nine anxiety and 12 depression PROMs used to assess psychological distress in HNC populations and mapped them against DSM-5 criteria for depression and anxiety. While DSM-5 criteria for anxiety and depression are established on the premise that the target population is physically well, we chose this model to appraise conceptual coverage of PROMs in this study for two reasons: (1) mental health disorders are said to occur in about 40% of patients diagnosed with cancer [91, 92] and the DSM is an evidence-based guide to the diagnosis of mental disorders; and, (2) most depression and anxiety measures identified have been developed and validated based on DSM criteria.

The content covered by these measures varied in terms of their relevance and appropriateness to HNC. Most anxiety measures focused on restlessness, muscle tension, disturbing thoughts and worry. Content areas not adequately addressed in identified measures included sleep disturbance (covered only by Duke-AD), fatigue, concentration and irritability. Similarly, depression measures revealed great content disparity. All identified measures addressed ‘depressed mood’ but only a few addressed ‘change in weight’.

For patients diagnosed with HNC, anxiety and depression extends beyond the completion of their treatment as side effects impact their everyday functioning [8]. Neurovegetative symptoms used to denote depression in non-cancer populations, such as change in weight, sleep disturbances and loss of appetite, are likely to be disease- or treatment-related in people diagnosed with cancer, and therefore not good indicators of depression in this population [93]. Hence, cognitive symptoms such as worthlessness or guilt, low self-esteem, depressed mood, concentration or indecisiveness need to be monitored to detect depression. Therefore, PROMs such as PHQ-9 and MDI, which have no or few items assessing neurovegetative symptoms may be more relevant and appropriate for use in HNC populations. For anxiety, one symptom that may confound HNC and its treatment is fatigue. As fatigue is an important indicator of clinical anxiety, it is essential to consider when reviewing content coverage of anxiety measures. Of the anxiety measures, SAS and STAI had the greatest content coverage against the diagnostic standard DSM-5 criteria. While we acknowledge avoiding symptoms potentially due to illness, we sought the most comprehensive coverage of anxiety and depression symptoms to ensure maximum sensitivity in detecting anxiety and depression, with the proviso that these would need to be clinically assessed to judge causation. Therefore we chose measures which included fewer neurovegetative symptoms, excellent conceptual coverage and good psychometric properties.

Appraisal of PROM psychometric properties determined the MDI and CDS had inadequate psychometric properties, and the QIDS-SR does not have established clinical cut-off scores for cancer populations. Hence, for depression, PHQ-9 and SDS are recommended for use in the HNC setting due to their comprehensive content coverage relevant to HNC and robust psychometric properties. For anxiety, the STAI has questionable predictive accuracy, discriminant and factorial validity, and failed to effectively differentiate between subjects with and without anxiety disorders [94, 95]. Furthermore, the primary purpose of this measure was to assess the severity of state and trait anxiety, with the trait scale overlapping with symptoms of depression [94, 96]. Hence, the SAS, which has good content coverage and psychometric properties, is recommended for use in the HNC setting to assess anxiety.

All PROMs identified in this study were developed to either measure anxiety or depression or both. Purposes of these measures varied only in the extent of assessment – whether they were developed as a screening tool or to assess the extent or severity of anxiety and depression. All measures had established clinical cut-off points for anxiety and depression for the general population, but only three anxiety measures and seven depression measures had cut-off points for cancer. Clinical cut-off scores have been determined using data from populations with severe emotional disorders and healthy subjects to classify the extent of a mental disorder based on a screening or outcome measure. However, these cut-off scores may not be appropriate for cancer populations where anxiety and depression can be a normal response to a traumatic life event. To differentiate clinical levels of anxiety and depression in cancer populations, distinct cut-off points need to be established in these populations [97]. Some depression measures (such as QIDS-SR, GDS-SF, Duke-AD, MDI and CDS) and anxiety measures (STAI, SAS, BAI, MASQ, Duke-AD, GAD-7) failed to report clinical cut-offs specifically for cancer populations. Clinical cut-off points ascertained exclusively for cancer populations would better discriminate between the presence and absence of clinical anxiety and depression, reducing the number of false positives in practice. This will enable healthcare providers to effectively assess the mental health of people with cancer under their care, identifying those requiring clinical intervention, and making appropriate referals.

According to previous studies, PROMs assessing anxiety and depression most commonly used in HNC populations were HADS, BDI, CES-D and QIDS-SR [10, 98]. However, all other identified measures except MASQ, Duke-AD, MDI and CES-D have been used in HNC populations at least once. Evaluation of criterion validity (determined using receiver operating characteristic curve) has been reported so far for CES-D, BDI and HADS [8, 27, 99, 100] with HNC populations.

### Limitations and implications

This systematic review was rigorously conducted, but has some limitations. First, only primary research published in English was included. Second, development and validation studies were hand-searched; it is possible some publications were missed. Third, the quality of individual studies was not reviewed, however, the focus of this systematic review was on PROMs and not primary study design and reporting quality.

This systematic review summarises available HNC-specific measures for assessing anxiety and depression, and provides a reliable source of evidence to guide measurement selection for research and clinical practice. Literature shows that there is a need for cross-cultural language translations of PROMs [101] and findings from this study offer a starting point in determining which PROMs may be suitable for cultural adaption and validation in HNC populations.

## Conclusions

To summarize, based on content mapping and appraisal of psychometric properties, SAS, SDS and PHQ-9 were considered most suitable for use in HNC populations to assess anxiety and depression. It is important to use PROMs for assessing anxiety and depression that capture a comprehensive set of subjective symptoms. This review highlights the importance of establishing disease-specific clinical cut-offs for common psychological variables such as anxiety and depression, to facilitate accurate diagnoses in cancer patients. It also explains how some symptoms of anxiety and depression can be confounded by those caused by the disease and its treatment.

## Supplementary information

**Additional file 1.**

## Data Availability

The data that support the findings of this study are available from the corresponding author upon reasonable request.

## Abbreviations

HNC: Head and neck cancer
PROM: Patient-reported outcome measure
DSM: Diagnostic and Statistical Manual
HRQOL: Health Related Quality of Life
COSMIN: COnsensus-based Standards for the selection of health Measurement Instruments
POD: Psycho-oncology database
GEM: Grid-enabled Measures Database
HADS: Hospital Anxiety and Depression Scale
STAI: State trait Anxiety Inventory
SAS: Zung Self Rating Anxiety Scale
BAI: Beck’s Anxiety Inventory
MASQ: Mood and Anxiety Symptom Questionnaire
DASS 21: Depression Anxiety Stress Scale 21
Duke-AD: Duke Anxiety Depression Scale
BSI-18: Brief Symptom Inventory – 18
GAD – 7: Generalized Anxiety Disorder – 7
QIDS-SR: Quick inventory of Depressive Symptomatology self-report
SDS: Zung Self-Rating Depression Scale
BDI: Beck’s Depression Inventory
CES-D: Center for Epidemiologic Studies Depression Scale
PHQ-9: Patient Health Questionnaire – 9
GDS-SF: Geriatric Depression Scale -Short Form
MDI: Major Depression inventory
CDS: Carroll Rating Scale for Depression
