# Supplementary material for: What are the optimal measures to identify anxiety and depression in people diagnosed with head and neck cancer (HNC): a systematic review
Source: J Patient Rep Outcomes. 2020 Apr 23;4:26. doi: 10.1186/s41687-020-00189-7 (PMC7181465; doi:10.1186/s41687-020-00189-7)
Supplement: Supplementary file 1 — Additional file 1. [file 41687_2020_189_MOESM1_ESM.docx]

Additional file 1

**Advanced search performed with truncation wherever necessary done on 05 September 2017 limiting years (2007 to 2017) and language (English only) combined using boolean operators (AND or OR). The search was repeated on 01 August 2019 limiting years (2017 to 2019) and on 14 January 2020 limiting years 2007 to 2020 (current) with updated key terms.**

**1. cancer.mp. or exp Neoplasms/**

**2. (head and neck).mp. [mp=title, abstract, original title, name of substance word, subject heading word, floating sub-heading word, keyword heading word, organism supplementary concept word, protocol supplementary concept word, rare disease supplementary concept word, unique identifier, synonyms]**

**3. (head or neck).mp. [mp=title, abstract, original title, name of substance word, subject heading word, floating sub-heading word, keyword heading word, organism supplementary concept word, protocol supplementary concept word, rare disease supplementary concept word, unique identifier, synonyms]**

**4. Nasopharyngeal Neoplasms/ or Nasopharynx/ or Nasopharyngeal Carcinoma/**

**5. Oropharyngeal Neoplasms/ or Oropharynx/ or Oropharyn*.mp.**

**6. Laryngeal Neoplasms/ or Laryn*.mp. or Larynx/**

**7. Hypopharyngeal Neoplasms/ or Hypopharynx/ or Hypopharyn*.mp.**

**8. nasal cavity.mp. or exp Nasal Cavity/**

**9. sinus.mp.**

**10. Salivary gland*.mp. or Salivary Glands/**

**11. throat.mp. or exp Pharynx/**

**12. lip.mp. or Lip/ or exp Lip Neoplasms/**

**13. Paranasal sinus.mp. or Paranasal Sinuses/**

**14. Carcinoma, Squamous Cell/ or Metastatic squamous cell.mp.**

**15. Oral cavity.mp. or exp Mouth/**

**16. Parotid Neoplasms/ or paroti*.mp.**

**17. Tonsil*.mp.**

**18. Tongue*.mp. or Tongue/ or Tongue Neoplasms/**

**19. Vocal cord*.mp. or Vocal Cords/**

**20. Pharynx/ or Pharyn*.mp. or Pharyngeal Neoplasms/**

**21. 2 or 3 or 4 or 5 or 6 or 7 or 8 or 9 or 10 or 11 or 12 or 13 or 14 or 15 or 16 or 17 or 18 or 19 or 20**

**22. 1 and 21**

**23. exp Anxiety/ or anxiety.mp. or exp Anxiety Disorders/ or Health anxiety.m.p or Health anxiety/**

**24. depression.mp. or Depression/**

**25. exp Depressive Disorder, Major/ or exp Depressive Disorder/ or depressive disorder.mp**

**26. psychological distress.mp.**

**27. distress.mp.**

**28. exp Stress, Psychological/ or stress.mp.**

**29. Mental Health/ or mental health.mp.**

**30. depersonalization.mp. or Depersonalization/**

**31. emotional exhaustion.mp.**

**32. low mood.mp.**

**33. psychological wellbeing.mp.**

**34. psychological symptoms.mp.**

**35. worry.mp.**

**36. psychological function*.mp.**

**37. 23 or 24 or 25 or 26 or 27 or 28 or 29 or 30 or 31 or 32 or 33 or 34 or 35 or 36**

**38. 22 and 37**

**39. limit 38 to (english language and yr="2007 -Current")**
